# Lactobacilli can attenuate inflammation in mouse macrophages exposed to polyethylene particles in vitro

**DOI:** 10.1186/s13104-018-3676-z

**Published:** 2018-08-08

**Authors:** Meera Esvaran, Patricia L. Conway

**Affiliations:** 1 Centre for Marine Bio-Innovation, The University of New South Wales, Sydney, NSW 2052 Australia; 2 NAFTEC, School of Chemical and Biomedical Engineering, Nanyang Technology University, Singapore, 637459 Singapore

**Keywords:** Ceridust 3165, Cytokine, Inflammation, TNF-α, Wear debris, Lactobacilli, RAW 246.7, Macrophages

## Abstract

**Objective:**

It is well established that polyethylene (PE) wear particles induce macrophage production of cytokines and mediators associated with the pathogenesis of inflammatory osteolysis. The objective of this study was to examine the potential of three Lactobacillus strains to attenuate the TNF-α cytokine response of macrophages exposed to Ceridust 3615 PE particles. An in vitro experimental model using the RAW 246.7 macrophage cell line and PE particles was utilized.

**Results:**

Lactobacillus strains were found to modulate the cytokines in a strain and dose specific manner. Only the *Lactobacillus acidophilus* strain that was tested was able to attenuate PE particle-induced TNF-α production by RAW 246.7 macrophages. This effect was independent of IL-10 cytokine levels since all three strains of lactobacilli yielded comparable levels of IL-10. It was concluded that some, but not all, Lactobacillus strains may be useful in reducing the risk of inflammatory osteolysis and that further studies in appropriate in vivo models are warranted. Furthermore, this in vitro model can be used to evaluate the inflammatory potential of new materials being tested for use as joint implants.

## Introduction

Of the 1.5 million joint arthroplasty operations that are performed annually worldwide, about 10% of patients develop inflammatory osteolysis and subsequent aseptic joint loosening within 15 years of the initial surgery. Central to the inflammatory process is the interaction of macrophages with debris from prosthetic implants. Polyethylene (PE) and polymethylmethacrylate wear particles generated from the surfaces of articulating prosthetic devices in total joint replacements have been implicated in the aseptic loosening of the prosthesis [1]. Wear particles are phagocytosed, but not digested by the macrophages, which leads to an increased production of pro-inflammatory cytokines and mediators such as IL-1, IL-6, IL-8 and TNF-α [2, 3]. Over production of these cytokines leads to inflammation, osteolysis, loosening of the prosthesis and ultimately in rejection of the implant [1]. The role of TNF-α in inflammatory osteolysis has been demonstrated [4]. Inhibition of TNF-α has been found to reduce wear particle induced osteolysis in both in vitro and animal models [5, 6].

Strains of lactobacilli have a ‘generally regarded as safe’ status and are used extensively in the food industry and as dietary supplements. An advantage of using lactobacilli is that oral administration can elicit systemic immune responses including modulation of pro-inflammatory cytokines [7–9]. Whole bacterial cells, their components and secreted factors of some lactobacilli strains can suppress the release of inflammatory cytokines such as TNF-α and IL-12 by macrophages [10, 11]. This paper examines the capacity of three Lactobacillus strains to modulate PE particle induced TNF-α in RAW 246.7 macrophages in vitro as a potential oral therapy for reducing the risk of inflammatory osteolysis.

## Main text

### Materials and methods

#### Bacterial strains and culture conditions

The *Lactobacillus* strains, *L. fermentum* PC1 (FII 511 400), *L. fermentum* PC2 (FII, 548, 700), and the *Lactobacillus acidophilus* (LA; FII 545 600) were obtained from the culture collection in the School of Biotechnology and Biomolecular Sciences, the University of New South Wales (UNSW). Lactobacilli were grown in de Mann Rogosa Sharpe broth in an anaerobic chamber maintained at 37 °C. Glycerol stocks of lactobacilli were inoculated (1%) into MRS broth, and propagated at appropriate conditions for 18 h prior to use as a primary culture. Secondary cultures were used for the experiments.

#### RAW 246.7 murine macrophage cell line and culture conditions

The macrophages were a kind gift from Dr. Carmel Quinn (UNSW). They were cultured and maintained in RPMI 1640 tissue culture medium supplemented with 10% fetal calf serum (FCS), 100 U/ml penicillin, 100 μg/ml streptomycin (culture medium). All tissue culture reagents were purchased from GIBCO (USA).

#### Ceridust 3615 polyethylene particles

Ceridust 3615; commercially available polyethylene (PE) particles were kindly provided by Professor William Walsh (UNSW, Australia). The mean particle size was 0.1–10 μm in diameter. Prior to use in experiments, the wear particles were subjected to 200 °C dry heat for 5 h, followed by incubation in 70% ethanol for 48 h, to remove contaminants, including endotoxins.

#### Endotoxin assay

The endotoxin levels for the PE particles were quantified using the ToxinSensor™ Chromogenic LAL Endotoxin Assay Kit (GenScript, USA) according to the manufacturer’s instructions. Briefly, a suspension was created by adding PE particles to the reagent water (provided in the kit). This suspension was vortexed vigorously for 10 min, then left at room temperature for 1 h. The suspension was then centrifuged at 1000×*g* for 10 min and the supernatant was tested. Known concentrations of *Escherichia coli* endotoxin standard solutions ranging from 0.1, 0.05, 0.025 and 0.01 EU/ml were used to generate a standard curve. The PE particles were found to contain less than 0.01 EU/ml of endotoxin.

#### Cytotoxicity assay

The toxicity of lactobacilli on the RAW 246.7 macrophages was examined using the CytoTox96^®^ Non-radioactive Cytotoxicity assay (Promega, USA) according to the manufacturer’s instructions. Briefly, the macrophages (1 × 10^4^ cells per well) were incubated for 4 h at 37 °C, 5% CO_2_ after which they were incubated with varying concentrations of lactobacilli cells for a further 4 h. Control wells had no added lactobacilli. Macrophage viability was expressed as the optical density (OD) of treated cells and was calculated as follows: cell viability (%) = (OD of treatment group ⁄ OD of control group) × 100.

#### Effect of lactobacilli on cytokine production by macrophages stimulated with PE particles

Due to their low density, PE particles float on top of the culture medium, thus limiting the contact between the macrophages and the PE particles. To maximise contact, the particles were seeded into soft agar and the macrophages were layered over them as previously described by Green et al. [12]. It has previously been calculated that 1 μm^3^ of the PE particles had a weight of 1 × 10^−9^ mg. A ratio of particle volume (μm)^3^:macrophage cell number of 10 was used. Briefly, the particles were suspended in culture medium supplemented with 30% FCS. This colloidal suspension was then mixed with a 1% (w/v) agarose solution (in culture medium) at a ratio of 2:1 and dispensed into wells of 48-well plates (200 μl/well; Greiner, Australia). The plates were centrifuged in a plate spinner at 800×*g* resulting in a superficial upper layer of PE particles. Macrophages were overlaid on the set gel (1 × 10^5^ cells per well) and the plates then placed in a CO_2_ incubator for 4 h to allow for the macrophages to adhere (5% CO_2_ at 37 °C). The growth medium was aspirated. Different doses of lactobacilli (10, 1 or 0.1 bacteria per macrophage) were added to the wells before incubation for 4 h. The bacterial cells were washed off and fresh culture medium was added and incubated for a further 12 h. Supernatant was collected for cytokine analysis.

#### Cell viability assay

At the end of the experiment, macrophage viability was assessed using the Trypan blue method. Media controls (macrophages cultured on gels without particles) and positive controls (macrophages stimulated with lipopolysaccharide (1 µg/ml; Sigma, Australia) in the absence of particles and lactobacilli) were included.

#### Cytokine assays

Cell culture supernatants were assayed for cytokine IL-10 using enzyme-linked immunosorbent assays (ELISA) with matched-antibody pairs and recombinant standards (Pharmingen, USA). The plates were read at 450 nm using the Bench Mark microplate reader (Bio-Rad, USA). The concentrations of the IL-10 in the samples were calculated using a standard curve.

Biologically active TNF-α in the culture supernatant was quantified using a bioassay employing WEHI-164 cells [13]. Concentrations of TNF-α were standardized using mouse recombinant TNF-α (BD Pharmingen, USA). The limit of detection of this assay was 0.0195 U/ml.

#### Statistics

The results presented are the average of the two experiments. The data are expressed as the means + standard error of mean (SEM) of triplicate wells. The experimental data was analysed by one two-way ANOVA using XLSTAT statistical software. The differences between the mean of groups were determined using TUKEY (HSD) test. P-values of less than 0.05 were considered statistically significant.

### Results and discussion

#### Effect of lactobacilli on cytokine production by macrophages stimulated with PE particles

It is well established that wear particles at the articulating surfaces of prosthetic implants are important causative agents in prosthetic osteolysis. TNF-α is one of the primary mediators released by macrophages stimulated by wear-particles and instrumental in the development of prosthetic osteolysis. PE particles used in this study have previously been shown to stimulate murine macrophages to produce TNF-α, IL-1 β, and IL-6 in vitro [2, 3].

**Fig. 1:**
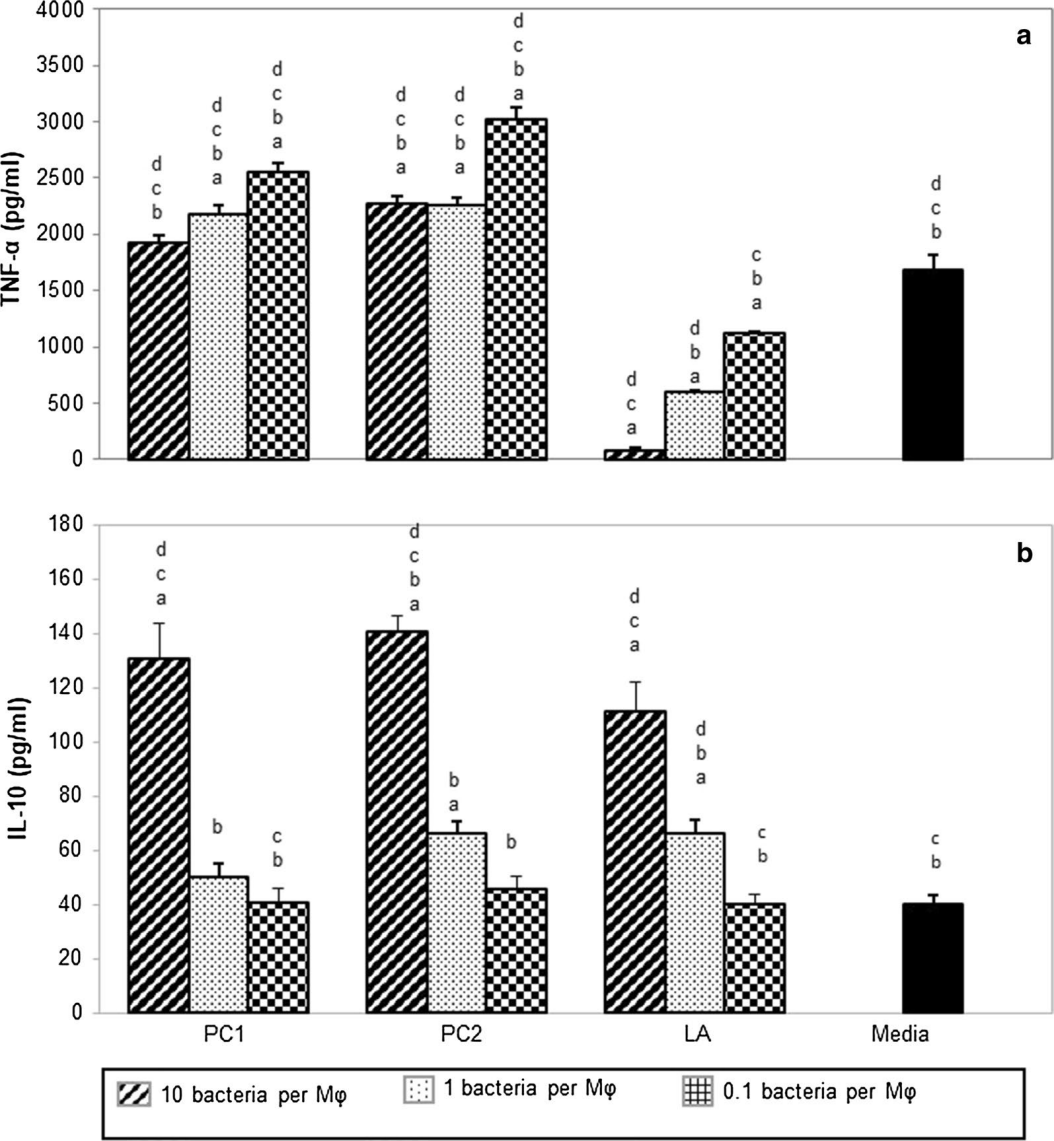
Effect of *Lactobacillus* strains PC1, PC2 and LA on PE particle induced TNF-α (**a**) and IL-10 (**b**) from macrophages. Macrophages were stimulated with PE particles for 4 h prior to the addition of different strains of lactobacilli at three doses (10, 1 or 0.1 bacteria per macrophage) followed by a further 12 h incubation. Data are presented as the mean + SEM of two experiments, each conducted in triplicate. ^a^P < 0.05, compared with PE particle control wells; ^b^P < 0.05, compared to high dose LA; ^c^P < 0.05, compared to medium dose LA; ^d^P < 0.05, compared to low dose LA. Control wells (dark) had PE stimulated macrophages cultured in media only

As can be seen in Fig. 1a, strain LA regulated PE particle induced TNF-α production by the macrophages in a dose dependent manner (Fig. 1a). All doses of LA significantly suppressed TNF-α production compared to the positive control wells exposed to PE particles (P < 0.0001). Neither PC1 nor PC2 strains suppressed TNF-α production by the macrophages. In fact, most of the doses of PC1 and PC2 significantly enhanced TNF-α production (P < 0.05). It was also noted that all doses of LA1 demonstrated significantly suppressed TNF-α production compared to most doses of strains PC1 and PC2 (P < 0.05).

Endotoxins can stimulate the release of pro-inflammatory cytokines by macrophages. We removed possible endotoxin contaminants by treating the PE particles with 70% ethanol prior to use in the study. The resulting particles had undetectable levels of endotoxin. It must be noted that the endotoxin assay employed may lack sensitivity [14] and therefore the possibility of very low levels of endotoxin contamination cannot be ruled out. However, the fact that only strain LA was able to attenuate TNF-α production by the PE stimulated macrophages is noteworthy, and independent of the possible presence of trace levels of endotoxin on the PE particles. This study reinforces the strain dependent immune effects of lactobacilli that have been reported other workers [10, 11].

Cytokine IL-10 is the most extensively studied of all anti-inflammatory cytokines. When IL-10 was first discovered, it was known as cytokine synthesis inhibitory factor due to its ability to inhibit multiple inflammatory cytokines and mediators including TNF-α [15]. Yang et al. reported a correlation between the protection conferred by viral IL-10 gene transfer in a murine model of wear particle induced osteolysis and the suppression of mRNA TNF-α expression [16]. All three lactobacilli strains demonstrated dose dependent ability to enhance IL-10 by the PE stimulated macrophages. As shown, all three strains of Lactobacillus produced comparable levels of IL-10 at the different doses. At the highest dose all strains induced significantly more IL-10 than the PE particles control wells (P < 0.001) (Fig. 1b). At the medium dose, strains PC2 and LA induced significantly more IL-10 than the PE particles control wells (P < 0.011 and 0.012 respectively) (Fig. 1b). However, as only the LA strain suppressed TNF-α production, it is possible that *L. acidophilus* regulates PE particles induced TNF-α through an IL-10 independent pathway. It is possible that the suppression of the pro-inflammatory cytokine, TNF-α, may be mediated by factors secreted by the lactobacilli that bind to, or interfere with the TNF-α signalling pathway. Molecules secreted by other lactobacilli strains have been shown to suppress TNF-α production by activated macrophages [17].

#### Effect of lactobacilli on TNF-α production by naïve macrophages

**Fig. 2:**
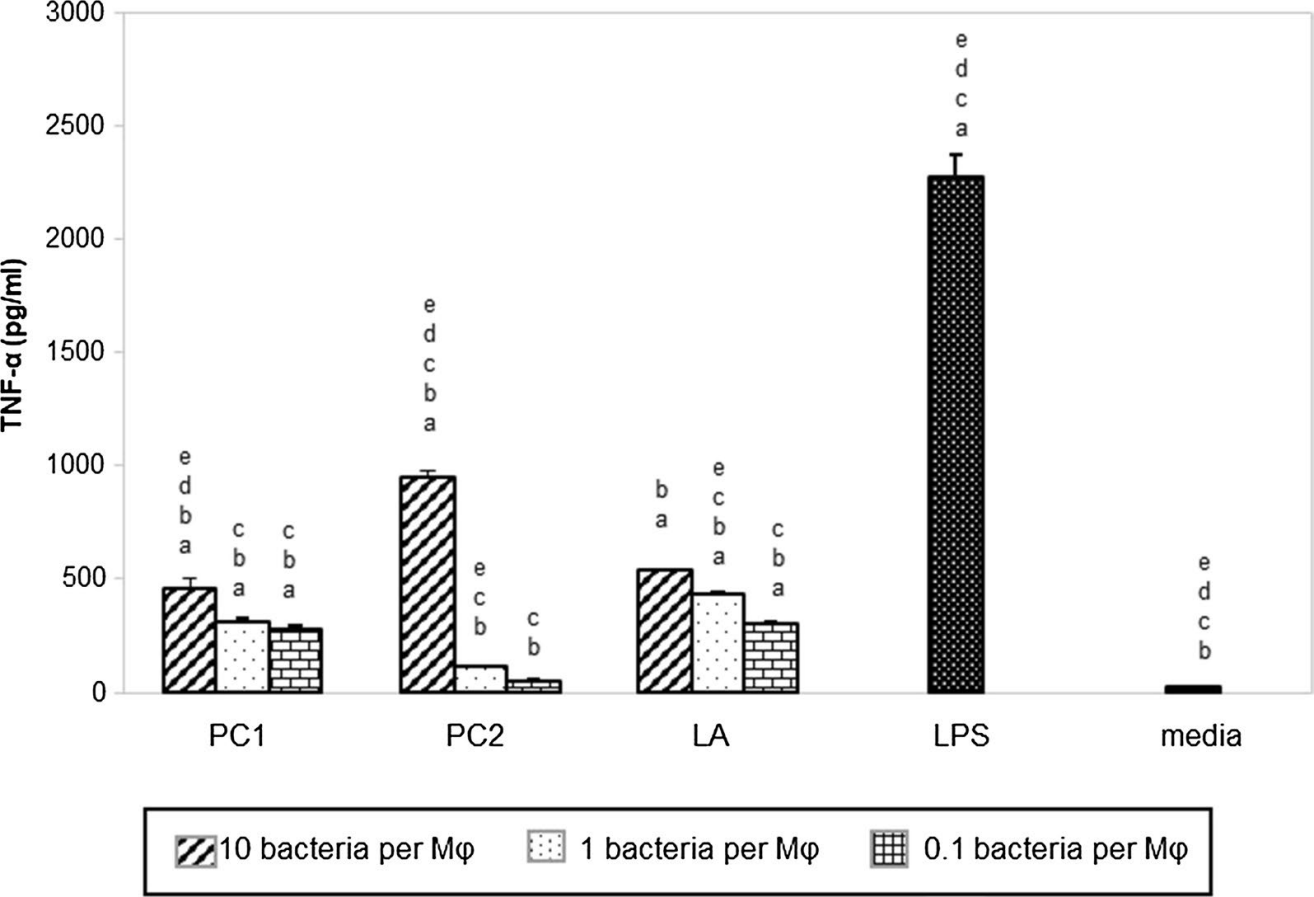
Levels of TNF-α production by naïve macrophages. Macrophages were cultured with different concentrations (10, 1 or 0.1 bacteria per MΦ) of three different strains of lactobacilli, namely PC1, PC2 and LA for 12 h. Positive control wells were treated with LPS (1 µg/ml) for 12 h. Control wells (dark) had macrophages cultured in media. Data are represented as mean + SEM of two pooled experiments. Each group had triplicate wells. ^a^P < 0.05, compared to media control; ^b^P < 0.001 compared with LPS control; ^c^P < 0.05, compared to high dose LA; ^d^P < 0.05, compared to medium dose LA; ^e^P < 0.05, compared to low dose LA

Some researchers express concerns that a bacteria or agent capable of significantly inhibiting PE particles induced TNF-α levels may also suppress TNF-α levels in general, leading to a state of immune suppression. Therefore, the capacity of the three lactobacilli strains to elicit TNF-α by naïve macrophages was examined (Fig. 2). A dose dependent effect was evident. At higher doses of lactobacilli, greater production of TNF-α by the macrophages was noted. In general, for each dose, all three lactobacilli produced comparable levels of TNF-α. Strains PC1 and LA (at all doses) and PC2 at the highest dose produced significantly more TNF-α than the medium control (P < 0.0001). However, all concentrations of lactobacilli at all doses produced significantly less TNF-α and significantly less than the LPS control (P < 0.0001). This demonstrates that the tested lactobacilli strains did not depress TNF-α production. They did enhance TNF-α production but not to the levels elicited by LPS.

#### Cell cytotoxicity

The incubation of the macrophages with increasing concentrations of the three Lactobacillus strains from 1 × 10^4^ to 1 × 10^10^ cells per well for 4 h did not result in decreased viability of the macrophages beyond baseline levels when assayed using the cytotoxicity assay. Cell viability was also examined at the end of each experiment. Cell viability of the treated wells was found to be comparable to the medium control wells. Therefore, it was concluded that the tested lactobacilli cells were not toxic to macrophages.

### Conclusion

It is shown that *L. acidophilus* LA attenuated PE particle induced TNF-α in macrophages in vitro. In addition, this inhibitory effect was found to be independent of IL-10. Strain LA has the potential to be used as an oral therapeutic agent in inflammatory osteolysis. However, further work needs to be done to ascertain the mode of action of LA in an appropriate in vivo model.

## Limitations

This is a preliminary paper where it was very exciting to see the differential expression of cytokine release by macrophages in response to stimulation by Ceridust 3165. At present, this observation is not backed by mechanistic data to explain how the *L. acidophilus* LA strain alone was able to suppress TNF-α production. Furthermore, this strain needs to be tested in an appropriate animal model to validate findings reported in this paper.

## Abbreviations

LA: *Lactobacillus acidophilus*
PC1: *Lactobacillus fermentum* PC1
PC2: *Lactobacillus fermentum* PC2
LPS: lipopolysaccharide
PE: polyethylene
